# Mechanisms of Spontaneous Climbing Fiber Discharge-Evoked Pauses and Output Modulation of Cerebellar Purkinje Cell in Mice

**DOI:** 10.3389/fncel.2017.00247

**Published:** 2017-08-22

**Authors:** Xian-Hua Jin, Hong-Wei Wang, Xin-Yuan Zhang, Chun-Ping Chu, Yuan-Zhe Jin, Song-Biao Cui, De-Lai Qiu

**Affiliations:** ^1^Key Laboratory of Cellular Function and Pharmacology of Jilin Province, Yanbian University Yanji, China; ^2^Department of Neurology, Affiliated Hospital of Yanbian University Yanji, China; ^3^Department of Endocrinology and Metabolism, Affiliated Zhongshan Hospital of Dalian University Dalian, China; ^4^Department of Physiology and Pathophysiology, College of Medicine, Yanbian University Yanji, China

**Keywords:** cerebellar Purkinje cell, whole-cell patch-clamp recording, complex spike (CS), after-hyperpolarization (AHP), simple-spike (SS), small conductance calcium-activated potassium channel (SK)

## Abstract

Climbing fiber (CF) afferents modulate the frequency and patterns of cerebellar Purkinje cell (PC) simple spike (SS) activity, but its mechanism is unclear. In the present study, we investigated the mechanisms of spontaneous CF discharge-evoked pauses and the output modulation of cerebellar PCs in urethane-anesthetized mice using *in vivo* whole-cell recording techniques and pharmacological methods. Under voltage-clamp recording conditions, spontaneous CF discharge evoked strong inward currents followed by small conductance calcium-activated potassium (SK) channels that mediated outward currents. The application of a GABA_A_ receptor antagonist did not significantly alter the spontaneous SS firing rate, although an AMPA receptor blocker abolished complex spike (CS) activity and induced significantly increased SS firing rates and a decreased coefficient of variation (CV) SS value. Either removal of extracellular calcium or chelated intracellular calcium induced a decrease in amplitude of CS-evoked after-hyperpolarization (AHP) potential accompanied by an increase in SS firing rate. In addition, blocking SK channels activity with a selective antagonist, dequalinium decreased the amplitude of AHP and increased SS firing rate. Moreover, we found repeated CF stimulation at 1 Hz induced a significant decrease in the spontaneous firing rate of SS, and accompanied with an increase in CV of SS in cerebellar slices, which was also abolished by dequalinium. These results indicated that the spontaneous CF discharge contributed to decreasing SS firing rate via activation of SK channels in the cerebellar PCs *in vivo* in mice.

## Introduction

Cerebellar Purkinje cells (PCs) exhibit spontaneous simple spike (SS) firing that is accompanied by irregular complex spike (CS) discharges *in vivo* (Ito, 1984). The bursting of SS firing is thought to be controlled by T-type calcium channels, as well as small conductance (SK) and large conductance (BK) calcium-activated potassium currents (Swensen and Bean, 2003). Under physiological conditions, the activation of BK channels requires both membrane depolarization and an increase in intracellular Ca^2+^, although activation of SK channels is not dependent on membrane voltage and can respond to Ca^2+^ entry only (Köhler et al., 1996; Vergara et al., 1998). Notably, SK channels contribute to an after-hyperpolarization (AHP) potential following bursts of action potentials and are involved in the regulation of spike firing frequency in some neurons (Stocker et al., 1999; Pedarzani et al., 2001). Additionally, dendritic ionic channels contribute to spontaneous SS activity in cerebellar slices (Womack and Khodakhah, 2002). For instance, dendritic Ca^2+^ influx via voltage-gated calcium channels (VGCC; Llinás and Sugimori, 1980; Usowicz et al., 1992) and P/Q-type calcium channels (Womack and Khodakhah, 2004) also contribute to SS burst firing in PCs *in vitro*.

Climbing fiber (CF) activation evokes distinctive high-frequency CSs followed by a pause of SS (Eccles et al., 1966), which suggests that CS activity is an important signal for the cerebellar cortex, conveying timing information from the outside to the cerebellar cortex (Welsh and Llinás, 1997) and triggering parallel fiber-PC synaptic plasticity (Hansel et al., 2001; Ito, 2001). The inhibition of CF discharges by inactivating or removing the inferior olive induces an increase in spontaneous SS frequency of PCs, revealing that CF input influences SS discharge in PCs *in vivo* (Colin et al., 1980; Montarolo et al., 1982; Cerminara and Rawson, 2004). Additionally, repetitive stimulus of CFs induces a progressive reduction and ultimately the total cessation of spontaneous SS activity (Colin et al., 1980; Demer et al., 1985). Under *in vitro* conditions, CF-induced changes in the PC SS firing rate could occur independently of network activation, although it is dependent on the level of intracellular Ca^2+^ (McKay et al., 2007). Repetitive CF discharges induce an increase in intracellular Ca^2+^ levels and enhance activation of Ca^2+^-dependent potassium channels, resulting in a change in SS firing properties (Hounsgaard and Midtgaard, 1989; Miyakawa et al., 1992; Maeda et al., 1999; McKay et al., 2007).

CF discharge evokes pause and AHP potential, which is assumed to be the result of recruited inhibitory interneurons by their CF inputs (Mathews et al., 2012), as well from activation of calcium-dependent SK-type K^+^ channels (Kakizawa et al., 2007). During downregulation of the unique isoform of SK channels, SK_2_ induces increased SS activity in PC intrinsic plasticity (Belmeguenai et al., 2010). Additionally, the inhibition or reduction of SK_2_ channels abolishes spike pause plasticity, which suggests that SK_2_ channels may be crucial to cerebellar information storage by altering PC output (Grasselli et al., 2016). Conversely, cerebellar cortical molecular layer interneurons (MLIs) activated by parallel fibers are involved in controlling PC output via powerful feed-forward inhibition (FFI) *in vitro* (Häusser and Clark, 1997; Mittmann et al., 2005). The inhibition of GABA_A_ receptor-mediated FFI decreases the PF stimulation-evoked number of SS firing in mouse cerebellar PCs (Wulff et al., 2009). However, single unit recordings from cerebellar PCs reveals and average SS firing frequency that is similar between GABA_A_ receptor γ_2_ subunit deleted (PC-Δγ_2_) and normal mice (Wulff et al., 2009).

Although the effects of CF activity on SS firing characteristics have been well established *in vitro* and *in vivo* by extracellular recording technique, the mechanisms of spontaneous CF discharge-evoked pauses and the modulation of cerebellar PCs in living animals remain unclear. In this study, we analyzed the mechanisms of spontaneous CS discharge modulation of SS activity in cerebellar PCs in urethane-anesthetized mice using *in vivo* whole-cell recording techniques and pharmacological methods. Results showed that either removal of extracellular calcium or chelated intracellular calcium induced a decrease in amplitude of CS-evoked AHP accompanied by an increase in SS firing rate. Inhibition of SK channels activity decreased AHP amplitude and increased the SS firing rate. These results indicated that spontaneous CF discharge contributed to decreasing the SS firing rate via activation of SK channels in cerebellar PCs in mice.

## Materials and Methods

### Anesthesia and Surgical Procedures

The anesthesia and surgical procedures have been previously described (Chu et al., 2011). In brief, the experimental procedures were approved by the Animal Care and Use Committee of Jilin University and were in accordance with the animal welfare guidelines of the U.S. National Institutes of Health. The permit number is SYXK (Ji) 2007-0011. Animals were housed under a 12-h light:12-h dark cycle with free access to food and water. Either male (*n* = 30) or female (*n* = 30) adult (6 to 8-week-old) HA/ICR mice were anesthetized with urethane (1.3 g/kg body weight i.p.). A watertight chamber was created and a 1–1.5-mm craniotomy was drilled to expose the cerebellar surface corresponding to vermis VI–VII. The brain surface was constantly superfused with oxygenated artificial cerebrospinal fluid (ACSF: 125 mM NaCl, 3 mM KCl, 1 mM MgSO_4_, 2 mM CaCl_2_, 1 mM NaH_2_PO_4_, 25 mM NaHCO_3_ and 10 mM D-glucose) using a peristaltic pump (Gilson Minipulse 3; Villiers, Le Bel, France) at 0.4 ml/min. Rectal temperature was monitored and maintained at 37.0 ± 0.2°C using body temperature equipment.

### Electrophysiological Recording in Urethane-Anesthetized Mice

**Figure 1:**
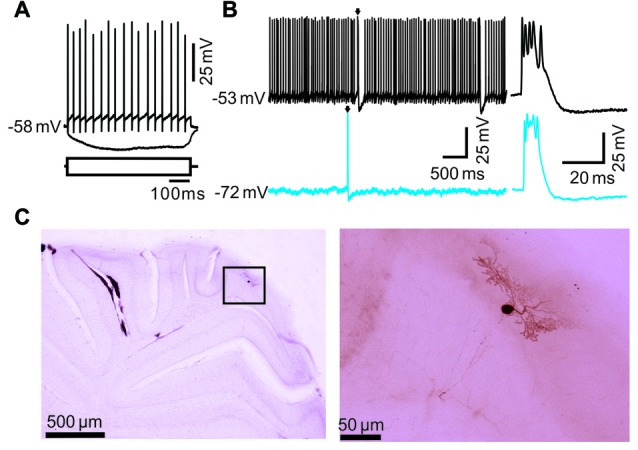
Whole-cell recordings showed the property of cerebellar Purkinje cell (PC) spontaneous activity in living mice. **(A)** Whole-cell patch-clamp recording from a soma of a PC in response to hyperpolarizing (–100 μA) and depolarizing (+100 μA) current pulses. **(B)** Under current-clamp, representative traces showing the spontaneous complex spike (CS; arrows) and simple spike (SS) activities when the membrane potential were clamped at −53 mV (*I* = 0 pA; upper) and −72 mV (lower). **(C)** The photomicrographs show the morphology of the cell, which is shown in **(A)**. The left column shows an overview of the location of the biocytin-labeled cell. The right column shows the detail of the biocytin-labeled cell.

*In vivo* whole-cell patch-clamp recordings from PC somas (*n* = 42 cells) were performed using an Axopatch-200B amplifier (Molecular Devices, Foster City, CA, USA) in the cerebellar cortical lobule vermis VIb from 42/60 mice. We failed to obtain whole-cell recordings from PC somas in a total of 18 mice, although one PC was recorded in 42 mice for further experiments. Signals of whole-cell recordings from PCs were acquired using a Digidata 1440 series analog-to-digital interface on a personal computer with Clampex 10.3 software. Patch-pipettes were made with a puller (PB-10; Narishige, Tokyo, Japan) from thick-wall borosilicate glass (GD-1.5; Narishige). Patch electrodes (4–6 MΩ) contained a solution of the following (in mM): potassium gluconate 120, HEPES 10, EGTA 1, KCl 5, MgCl_2_ 3.5, NaCl 4, biocytin 8, Na_2_ATP 4 and Na_2_GTP 0.2 (pH 7.3 with KOH, osmolarity adjusted to 300 mOsm). For BAPTA experiments, EGTA was replaced with 10 mM BAPTA. The whole-cell recordings from PC somas were performed at depths of 250–300 μm under the pia mater membrane, and identified by regular spontaneous SS accompanied with irregular CS (Chu et al., 2011). The series resistances were in a range of 10–40 MΩ and compensated by 80%. Membrane voltage and current were filtered at 2 kHz and digitized at 20 kHz. Membrane resistance (*R*_m_) was determined by applying −100 pA square pulses (600 ms) from a mean membrane potential of 60 (±0.14 mV; *n* = 42) under current-clamp recording conditions (Figure 1A). The *R*_m_ was calculated from steady-state membrane potential of five responses. Spontaneous activity was calculated from a train of interspike intervals recorded for 100 s. In addition, the whole-cell recordings from PC dendrites were identified by the attenuated backpropagating of Na^+^ action potentials (Chu et al., 2011), which were excluded from this study.

### Electrophysiological Recording in Cerebellar Slices

Cerebellar slices preparation has been previously described (Qiu and Knöpfel, 2007). Adult mice were deeply anesthetized with halothane and decapitated quickly. The cerebellum was dissected and placed in ice-cold ACSF bubbled with 95% O_2_/5% CO_2_. The sagittal slices of cerebellar cortex (250 μm thick) were prepared using a Vibratome (VT 1200s, Leica, Nussloch, Germany). The slices were incubated for ≥1 h in a chamber filled with 95%O_2_/5% CO_2_ equilibrated ACSF at room temperature (24–25°C) prior to recording.

Whole-cell patch-clamp recordings from PC somas in cerebellar slices visualized using a 60× water-immersion lens through a Nikon microscopy (Eclipse FN1, Nikon Corp., Tokyo, Japan). Patch pipette resistances were 5–7 MΩ in the bath, with series resistances in the range of 10–20 MΩ. Membrane potentials and/or currents were monitored with an Axopatch 700B amplifier (Molecular Devices, Foster City, CA, USA), filtered at 5 kHz, and acquired through a Digidata 1440 series analog-to-digital interface on a personal computer using Clampex 10.4 software (Molecular devices, Foster City, CA, USA). For CF electrical stimulation, current pulses (0.2 ms, 100 μA) at 1 Hz were delivered through a glass electrode. The stimulating electrode containing ACSF (0.1–0.5 MΩ) was placed in the granule cell (GC) layer of the cerebellar cortex for CF stimulation.

### Drug Application

The reagents, which included BAPTA, 1,2-bis(2-aminophenoxy)ethane-N,N,N′,N′- tetraacetic acid, gabazine (SR95531), hydrobromide (6-imino-3-(4-methoxyphenyl)-1(6H)- pyridazinebutanoic acid hydrobromide), NBQX, (2,3-dioxo-6-nitro-1,2,3,4-tetrahydrobenzo [f]quinoxaline-7-sulfonamide) and dequalinium, were purchased from Sigma-Aldrich (Shanghai, China). The drugs were dissolved in ACSF, and directly applied to the cerebellar surface using a peristaltic pump (0.5 ml/min) for 10 min.

### Data Analysis

The electrophysiological data were analyzed using Clampfit 10.3 software. Values are expressed as mean ± SEM. One-way ANOVA and Mann-Whitney-Wilcoxon test (SPSS software; Chicago, IL, USA) was used to determine the level of statistical significance between groups of data. *P*-values < 0.05 were considered to indicate a statistically significant difference between experimental groups.

## Results

### Properties of Spontaneous CS Activity of Cerebellar PCs *In Vivo* in Mice

In this study, a total 42 cerebellar PCs were recorded in the cerebellar cortex vermis VIb using *in vivo* whole-cell patch-clamp recording technique in urethane-anesthetized mice (Chu et al., 2011, 2012). According to previous studies (Ito, 1984; Chu et al., 2011; Liu et al., 2016), the PCs were identified by regular SS firing accompanied by irregular CS activity. Under current-clamp conditions (*I* = 0), the mean membrane potential was −52.6 ± 0.37 mV (*n* = 42 cells), and the mean frequency of SS firing was 37.5 ± 2.8 Hz (*n* = 42 cells). The PCs also exhibited an irregular CS firing at a range of 0.01–1.72 Hz (*n* = 42 cells), accompanied by a SS pause with a mean value of 88.7 ± 7.6 ms (*n* = 42 cells). We also estimated membrane resistance (*R*_m_) of the PCs by injecting square pulses (−100 pA, 600 ms) under current clamp recording conditions (Figure 1A). The mean *R*_m_ value of PCs was 150.3 ± 21.7 MΩ (*n* = 42 cells). Injection of bias currents maintained at PCs at −70 mV by inhibiting spontaneous SS activity, but did not prevent spontaneous CS activity (Figure 1B). The photomicrographs showed that the morphology of the recorded neuron was a PC (Figure 1C).

**Figure 2:**
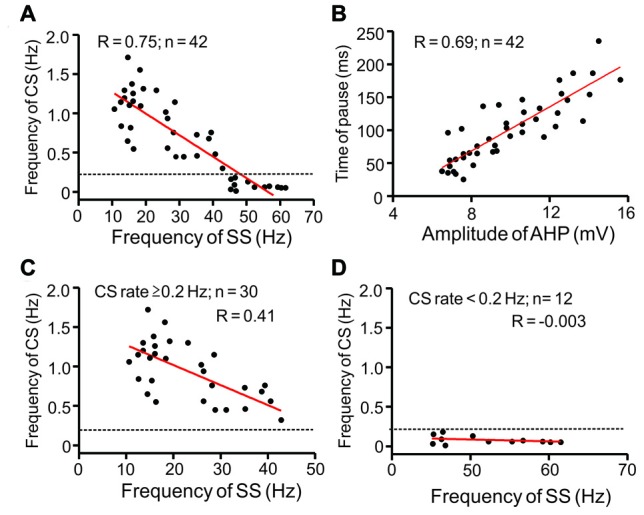
Scatter graphs show the relationship between CS and SS frequencies, amplitude of after-hyperpolarization (AHP) and time of pause. **(A)** Relationship between frequency of CS and SS rate. **(B)** Relationship between pause and AHP amplitude. **(C)** Relationship between high CS discharge rate (≥0.2 Hz; *n* = 30 cells) and SS rate. **(D)** Relationship between low CS discharge rate (<0.2 Hz; *n* = 12 cells) and SS rate. Note that the spontaneous CS firing rate negatively correlated with SS frequency, especially in PCs expressed the high CS discharge rate.

Furthermore, we analyzed the relationship between CS and SS frequencies, AHP amplitude, and time of pause. As shown in Figure 2, the spontaneous CS firing rate negatively correlated with SS frequency (Figure 2A; *R* = 0.75, *P* < 0.001), whereas time of pause positively correlated with AHP amplitude (Figure 2B; *R* = 0.69, *P* < 0.001) under current-clamp conditions. Notably, the high CS discharge rate (≥0.2 Hz) significantly correlated with frequency of SS activity (Figure 2C; *R* = 0.41, *P* = 0.003; *n* = 30 cells), although the low CS firing rate (<0.2 Hz) did not significantly correlate with frequency of SS firing (Figure 2D; *R* = −0.003, *P* = 0.35; *n* = 12 cells). These results indicated that an increase in CS firing rate accompanied a decrease in SS firing frequency in cerebellar PCs in mice.

### Effect of Spontaneous CS Activity on SS Firing Rate in Cerebellar PCs

**Figure 3:**
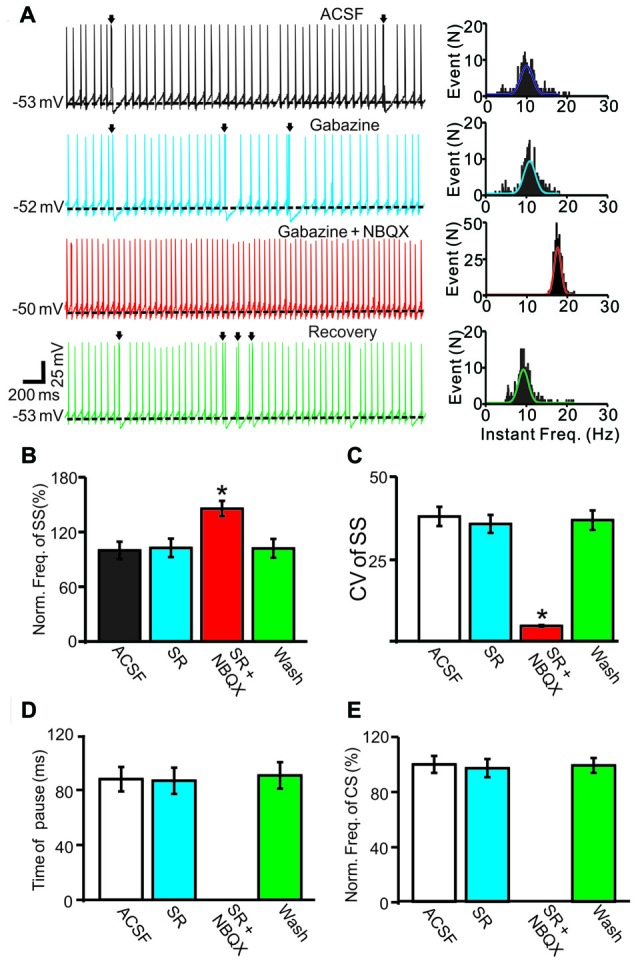
Effect of spontaneous CS activity on SS firing rate in cerebellar PCs. **(A)** Representative traces (left) and instant frequency (right) showing the spontaneous activity from of a PC in treatments: artificial cerebrospinal fluid (ACSF), gabazine (50 μM), gabazine + NBQX (250 μM) and recovery (washout; Arrows indicated CSs). **(B)** Summary of data showing the normalized SS firing rate of each treatment. **(C)** Pooled data showing the coefficient of variation (CV) values of SS in each treatment. **(D)** Bar graph showing the normalized pause of SS in each treatment. **(E)** Summary of data showing the normalized CS firing rate in each treatment. Note that application of AMPA receptor antagonist, NBQX completely blocked the CS activity accompanied with a significant increase in the spontaneous firing rate of SS. *P < 0.05; *n* = 6 cells.

Because CS discharge may affect PC SS activity through MLIs networks (Barmack and Yakhnitsa, 2003), we employed the GABA_A_ receptor antagonist gabazine (SR95531, 20 μM) to block MLI-mediated GABAergic inputs. In the presence of gabazine, the mean frequency of SS firing was 102.6 ± 10.1% of baseline (ACSF: 99.9 ± 9.5%; *F* = 0.09, *P* = 0.92; ANOVA; *n* = 6 cells; Figures 3A,B), and the coefficient of variation (CV) values of SS was 35.6 ± 2.7, which was similar to baseline (ACSF: 37.9 ± 2.9; *P* = 0.38; Mann-Whitney-Wilcoxon test; *n* = 6 cells; Figures 3A,C). However, additional application of the AMPA receptor antagonist NBQX (50 μM) induced a significant increase in SS firing rate and a decrease in CV value. In the presence of the mixture of gabazine and NBQX, the mean frequency of SS firing was 145.6 ± 8.4% of baseline (ACSF: 99.9 ± 9.5%; *F* = 8.3, *P* = 0.016; *n* = 6 cells; Figures 3A,B), and the CV values of SS was 4.6 ± 0.3, which was significantly less than baseline (ACSF: 37.9 ± 2.9; *P* < 0.0001; Mann-Whitney-Wilcoxon test; *n* = 6 cells; Figures 3A,C). In the presence of gabazine, the mean pause was 86.4 ± 9.5 ms, which was similar to control conditions (ACSF: 197.6 ± 8.9 ms; *F* = 0.07, *P* = 0.79; Mann-Whitney-Wilcoxon test; *n* = 6 cells; Figures 3A,C), and the normalized frequency of CS was 97.3 ± 6.6% of baseline (ACSF: 100.0 ± 6.2%; *F* = 0.074, *P* = 0.73; ANOVA; *n* = 6 cells; Figures 3A,D). Application of the mixture of gabazine and NBQX abolished CS discharge (Figures 3A,E), and no pause was observed (Figures 3A,D). These results indicated that the MLI networks contributed less to spontaneous SS firing activity, and CS discharge contributed to a decreased SS firing rate of cerebellar PCs *in vivo*.

**Figure 4:**
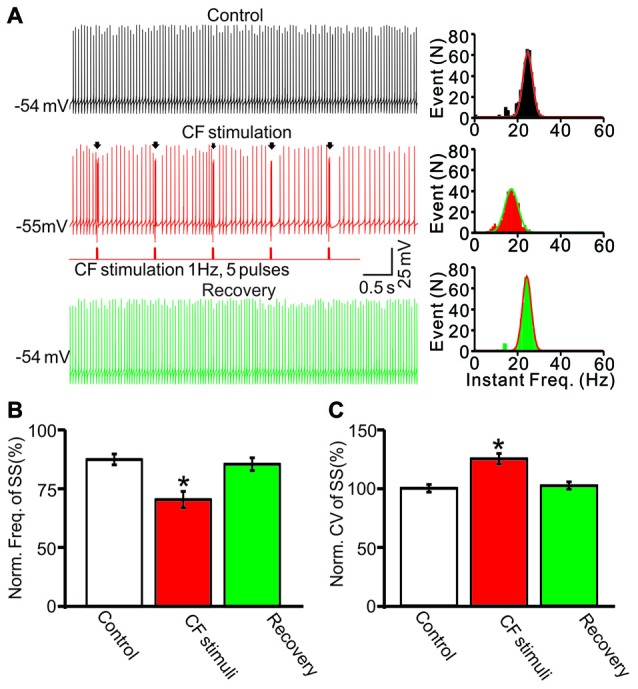
Stimulation of climbing fiber (CF) evoked CS activity induced a decrease in SS firing rate in cerebellar slices. **(A)** Left panel, representative traces showing the spontaneous activity of a PC under control conditions (control), during the CF stimulus (CF stimulation; 0.2 ms, 100 pA; 1 Hz, 5-pulse) and recovery. Arrows indicated the evoked-CSs. Right panel shows the instant frequencies of SS firing shown in the left panel. **(B)** Summary of data showing the normalized SS firing rate under control conditions (control), during the CF stimulus and recovery. **(C)** Bar graph showing the normalized CV of SS firing under control conditions (control), during the CF stimulus and recovery. Note that repeated CF stimulation at 1 Hz induced a significant decrease in the spontaneous firing rate of SS, and accompanied with an increase in CV of SS. *P < 0.05 vs. control; *n* = 10 cells.

Furthermore we examined the effect of CS discharge on SS firing rate by electrical stimulation of CF input in cerebellar slices. As shown in Figure 4, repeated CF stimulation at 1 Hz induced a significant decrease in the spontaneous firing rate of SS, the mean frequency of SS firing was 72.7 ± 2.6% of baseline (ACSF: 99.9 ± 1.7%; *F* = 259, *P* < 0.0001; ANOVA; *n* = 10 cells; Figures 4A,B). In addition, the mean CV value of SS was increased to 125.1 ± 4.4% of baseline (ACSF: 100.0 ± 3.2%; *F* = 70.2, *P* = 0.006; ANOVA; *n* = 10 cells; Figures 4A,C) during the repeated CF stimulation. These results indicated that CS discharge induced a significant decrease in the spontaneous firing rate of SS, and accompanied with an increase in CV of SS in cerebellar slices.

### Ionic Mechanism of Spontaneous CS Activity Depressed SS Firing Rate in Cerebellar PCs

**Figure 5:**
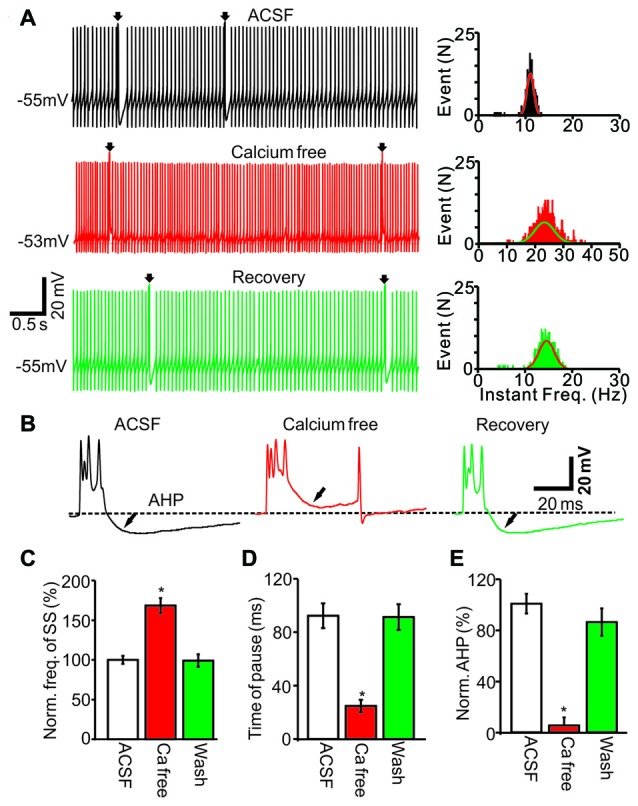
CS activity decreased SS firing rate was dependent on the extracellular calcium concentration. **(A)** Representative traces (left) and instant frequency (right) showing the spontaneous activity from of a PC in treatments of ACSF, calcium free and recovery (washout; arrows indicated CSs). **(B)** Enlarged CS traces shown in **(A)**. **(C)** Summary of data showing the normalized SS firing rate in treatments of ACSF, calcium free and recovery (washout). **(D)** Bar graph showing the normalized pause of SS in each treatment. **(E)** Summary of data showing the normalized amplitude of AHP in each treatment. Note that removing extracellular calcium induced a significant increase in the spontaneous firing rate of SS, and accompanied with decreases in pause of SS and AHP amplitude. *P < 0.05; *n* = 6 cells.

To understand the ionic mechanisms of how CF discharge modulates the SS firing rate, we examined whether the CS activity-induced decrease in SS firing rate was dependent on extracellular and intracellular calcium concentrations. We prepared a calcium-free extracellular solution by replacing calcium with an equal concentration of magnesium. Perfusion of calcium-free ACSF induced an increase in the SS firing rate (Figure 5A) accompanied by a decrease in AHP amplitude (Figure 5B). The application of a calcium-free solution resulted in an SS frequency of 168.3 ± 9.6% of baseline (ACSF: 100.0 ± 5.2%; *F* = 26, *P* = 0.0007; ANOVA; *n* = 6 cells; Figures 5A,C) and a pause mean time of 24.5 ± 4.5 ms, which was significantly shorter than control conditions (ACSF: 61.3 ± 9.1 ms; *P* < 0.001; Mann-Whitney-Wilcoxon test; Figures 5A,D). Additionally, the application of calcium-free ACSF decreased the AHP amplitude to 5.2 ± 6.4% of baseline (ACSF: 100.0 ± 7.7%; *F* = 316.5, *P* < 0.0001; ANOVA; *n* = 6; Figures 5A,E). These results showed that the removal of extracellular calcium decreased the CS-evoked AHP amplitude and increased the SS firing rate, suggesting that the CS activity-decreased SS firing rate was dependent on the extracellular calcium influx into cerebellar PCs.

**Figure 6:**
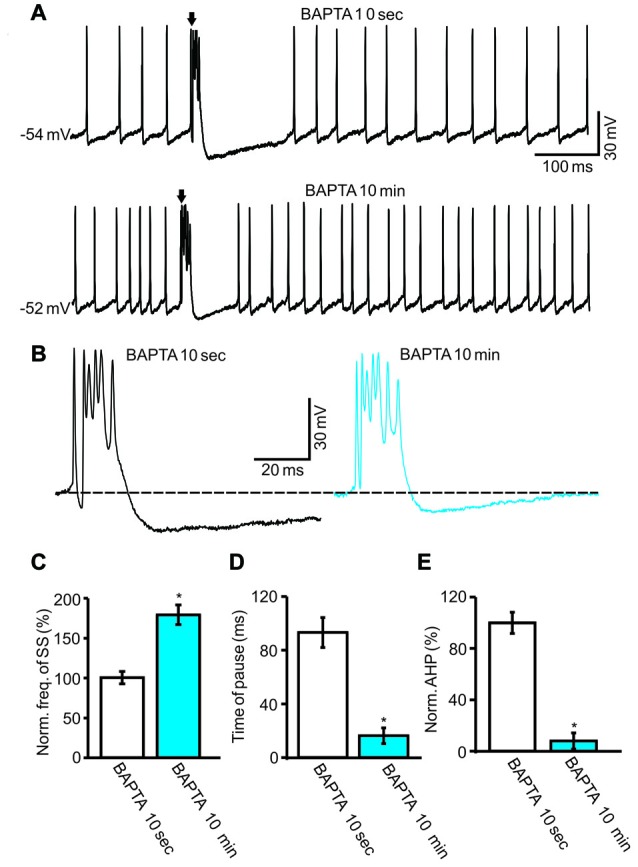
Effect of CS activity on SS firing rate required the intracellular calcium level. **(A)** Under current-clamp, representative traces showing the spontaneous CS and SS activities of a PC recorded with a BAPTA-including internal solution at 10 s and 10 min after the whole-cell configuration. **(B)** Enlarged traces of the CS waveforms shown in **(A)**. **(C–E)** Summary of data showing the normalized SS firing rate **(C)**, pause of SS **(D)**, amplitude of AHP **(E)** at 10 s and 10 min after the whole-cell configuration. Note that chelating intracellular calcium induced a significant increase in the spontaneous firing rate of SS, and accompanied with decreases in pause of SS and amplitude of AHP. *P < 0.05; *n* = 6 cells.

Furthermore, we examined whether intracellular calcium was required for CS activity-induced inhibition of the SS firing rate. We used a high concentration of fast-mobile Ca^2+^ buffer BAPTA (10 mM) in the pipette solution and observed a change in spontaneous activity as BAPTA diffused throughout the cytoplasm (Naraghi and Neher, 1997). The BAPTA-included internal solution significantly increased the SS spike firing rate after the whole-cell configuration (Figure 6A). After 10 min of whole-cell configuration, the normalized SS firing rate was 178.6 ± 12.3% of baseline (ACSF: 100.0 ± 7.6%; *F* = 215.7, *P* < 0.0001; ANOVA; *n* = 6 cells; Figures 6A,C). In addition, the application of intracellular BAPTA decreased the pause of SS (Figure 6A) and the amplitude of AHP (Figure 6B). After 10 min of whole-cell configuration, the mean time of pause was 16.2 ± 5.9 ms, which was significantly shorter than control conditions (ACSF: 93.6 ± 11.2 ms; *P* < 0.001; Mann-Whitney-Wilcoxon test; *n* = 6 cells; Figures 6A,D), and the AHP amplitude decreased to 7.9 ± 6.2% of baseline (ACSF: 100.0 ± 8.2%; *F* = 275.2, *P* < 0.0001; ANOVA; *n* = 6 cells, Figures 6B,E). Moreover, the normalized frequency of CS was 98.7 ± 3.5% of baseline (ACSF: 100.0 ± 6.4%; *F* = 0.065, *P* = 0.76; ANOVA; *n* = 6 cells; not shown). These results showed that chelating intracellular calcium failed to affect the spontaneous CS firing rate, but induced a significant increase in the spontaneous firing rate of SS, which was accompanied by a decreased pause of SS and AHP amplitude.

**Figure 7:**
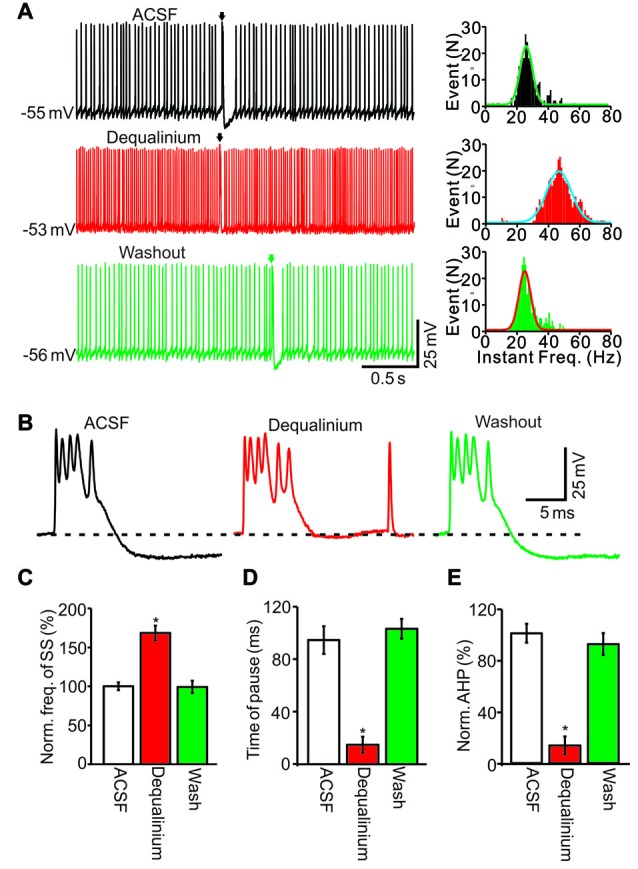
SK channels activity was involved in CS induced the decrease in SS firing rate. **(A)** Representative traces (left) and instant frequency (right) showing the spontaneous activity from of a PC in treatments of ACSF, dequalinium (10 μM) and washout (Arrows indicated CSs). **(B)** Enlarged CS traces shown in **(A)**. **(C)** Summary of data showing the normalized SS firing rate in treatments of ACSF, dequalinium and washout. **(D)** Bar graph showing the normalized pause of SS in each treatment. **(E)** Summary of data showing the normalized amplitude of AHP in each treatment. Note that blockade of SK channels induced a significant increase in the spontaneous firing rate of SS, and accompanied with decreases in pause of SS and AHP amplitude. *P < 0.05; *n* = 6 cells.

We then tested whether SK channel activity was involved in the CS-induced decrease in SS firing rate. Similar to the perfusion of calcium-free solution, blocking SK channel activity with the selective antagonist dequalinium (10 μM; Dunn, 1994), induced an increase in SS firing rate (Figure 7A) accompanied by a decreased AHP amplitude (Figure 7B). In the presence of dequalinium, the SS frequency was 176.3 ± 8.5% of baseline (ACSF: 100.0 ± 6.7%; *F* = 28, *P* = 0.0008; ANOVA; *n* = 6 cells; Figures 7A,C), and the mean pause was 14.7 ± 6.2 ms, which was significantly shorter that control conditions (ACSF: 94.5 ± 10.6 ms; *P* < 0.001; Mann-Whitney-Wilcoxon test; *n* = 6 cells; Figures 7A,D). Application of dequalinium decreased the AHP amplitude to 13.5 ± 6.9% of baseline (ACSF: 100.0 ± 7.7%; *F* = 217, *P* < 0.0001; ANOVA; *n* = 6 cells; Figures 7A,E). These results indicated that inhibition of SK channels induced a significant increase in the spontaneous firing rate of SS, which was accompanied by a decreased pause and AHP amplitude.

**Figure 8:**
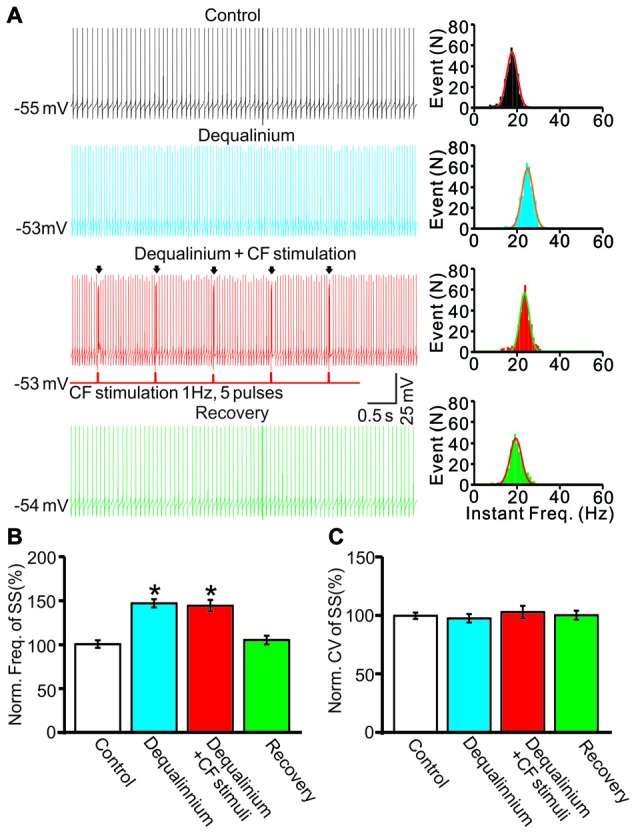
Stimulation of CF evoked CS activity failed to decrease SS firing rate of PCs in the presence of SK channel blocker. **(A)** Left panel, representative traces showing the spontaneous activity of a PC under control conditions (control), in the presence of SK channel blocker (dequalinium 10 μM), dequalinium + CF stimulus (Dequalinium + CF stimulation; 0.2 ms, 100 pA;1 Hz, 5-pulse) and recovery. Arrows indicated the evoked-CSs. Right panel shows the instant frequencies of SS firing shown in the left panel. **(B)** Summary of data showing the normalized SS firing rate under control conditions (control), dequalinium, dequalinium + CF stimulation and recovery. **(C)** Bar graph showing the normalized CV of SS firing under control conditions (control), dequalinium, dequalinium + CF stimulation and recovery. Note that SK channel blocker abolished the CF stimulation-induced effect on the spontaneous firing activity. *P < 0.05 vs. control; *n* = 7 cells.

In addition, we examined the effect of repeated CF stimulation at 1 Hz on SS activity in the presence of dequalinium (10 μM). Application of dequalinium induced an increase in SS firing rate (Figure 8A), the mean frequency of SS firing was 146.3 ± 4.8% of baseline (ACSF: 99.9 ± 4.2%; *F* = 204, *P* < 0.001; ANOVA; *n* = 7 cells; Figures 8A,B), but without effect the mean value of CV (Figure 8C). Importantly, in the presence of dequalinium, CS activity at 1 Hz failed to induce a decrease in SS firing rate, the mean frequency of SS firing was 143.7 ± 6.6% of baseline (ACSF: 99.9 ± 4.2%; *F* = 204, *P* < 0.001; ANOVA; *n* = 7 cells; Figures 8A,B), which was similar with dequalinium alone (146.3 ± 4.8% of baseline; *P* = 0.75; ANOVA; *n* = 7 cells; Figures 8A,B). These results indicated that SK channel blocker abolished the CF stimulation-induced effect on the spontaneous firing activity in cerebellar slices.

## Discussion

Results from the present study demonstrated that spontaneous CS discharge was responsible for an evoked AHP potential and contributed to decreasing SS firing rate in cerebellar PCs *in vivo*. In the presence of NBQX, blocking CF input, and in the presence a selective SK channel antagonist the SS firing rate of PCs increased. These results suggested that the spontaneous CS discharge contributed to decreasing the SS firing activity via activation of SK channels in cerebellar PCs in mice.

### Cerebellar PCs Exhibited Regular Spontaneous SS Firing Activity

The PC is the focus of computation in the cerebellar cortex, providing the sole output from the cerebellar cortex to the deep cerebellar nuclei (Ito, 1984). The tonic SS activity of cerebellar PCs has been suggested to be generated by intrinsic conductance, but that was modulated by parallel fiber and CF excitatory inputs (Häusser and Clark, 1997). Under *in vivo* conditions, cerebellar PCs exhibited SS firing at rates of 10–150 Hz, which was modulated by sensory stimuli such as tactile or retinal slip stimulation (Armstrong and Rawson, 1979; Ito, 1984; Ojakangas and Ebner, 1992; Ebner, 1998). The cerebellar PCs exhibit bistability of membrane potential and spike output, which could be induced by sensory stimulation or injuction of hyperpolarizing currents, and sometimes shown the switch between a depolarized and hyperpolarized state (Loewenstein et al., 2005; Schonewille et al., 2006; Witter and De Zeeuw, 2015). Additionally, MLIs play a critical role in modulating PC output. Stellate-type MLIs create synapses with PC dendrites, which block parallel fiber excitatory inputs onto PCs, although basket-type molecular interneurons project to the initial axonal segment and initial perisomatic inhibition of PCs. It was recently demonstrated that blocking FFI results in a significant increase in SS firing rates of cerebellar PCs in awake mice, suggesting that FFI contributes to shaping PCs output (Jelitai et al., 2016). Our present results were consistent with previous results (Santamaria et al., 2007; Chu et al., 2011), showing that the pharmacological blockade of GABAergic inhibition failed to increase the SS firing rate of cerebellar PCs under anesthetized conditions. We believe that these contradictory results are due to the type of anesthesia that was used. Urethane depresses neuronal excitability by activating barium-sensitive potassium leak conductance without affecting glutamate-mediated excitatory synaptic transmission or GABAA/B-mediated inhibitory synaptic transmission (Sceniak and Maciver, 2006). Under urethane-anesthetized conditions, the MLIs exhibited a spontaneous spike firing at a range of 0.08–4.26 Hz (Chu et al., 2012), and PCs fired at a mean frequency of 37.5 Hz (Chu et al., 2011; Liu et al., 2016; present data). Conversely, cerebellar PCs fired at a mean frequency of 61.9 Hz, and MLIs fired at 10–30 Hz in the absence of movement in awake mice (Chen et al., 2016; Jelitai et al., 2016). Therefore, urethane anesthesia likely depresses the activity of PC and MLI, as well reduces the synaptic input to PCs than the awake situation.

The ionic mechanisms of SS firing activity have been well demonstrated in cerebellar PCs *in vitro*. The intrinsic SS firing activity of PCs is primarily driven by resurgent Na^+^ channels, which contribute to repetitive firing by briefly reopening during the latter phase of action potential repolarization (Raman and Bean, 1997). However, the previous study illustrated that the removal of Ca^2+^ from extracellular solution resulted in more irregular firing behavior and a higher SS firing rate in PCs than under control conditions (Llinás and Sugimori, 1980). Therefore, the Ca^2+^ influx in cerebellar PCs via various Ca^2+^ channels near the membrane potential, at rest or hyperpolarization, is presumed to play a critical role during intrinsic SS firing activity (Llinás et al., 1989). Ca^2+^ influx modulates the intrinsic SS firing, which is presumed to take place following activation of Ca^2+^-activated K^+^ channels, such as SK and BK channels (Crepel and Penit-Soria, 1986; Raman and Bean, 1999; Swensen and Bean, 2003). However, the SK and BK channels play a unique role in shaping the electrical properties of the PC; SK channels influence the baseline firing frequency and BK channels influence the regulation of action potential shape and modulation of the CF response (Edgerton and Reinhart, 2003). Under physiological conditions, SK channels contribute to an AHP potential following bursts of action potentials, and are involved in the regulation of spike firing frequency in cerebellar PCs (Pedarzani et al., 2001). Following the inhibition of SK channels, PCs are unable to maintain a stable, tonic, firing pattern and instead oscillate between high frequency bursts and periods of quiescence (Edgerton and Reinhart, 2003). Additionally, PC dendrites express a high density of VGCCs and generate prominent dendritic calcium spikes (Llinás and Sugimori, 1980; Usowicz et al., 1992). Therefore, dendritic VDCCs significantly contribute to spontaneous SS firing activity (Womack and Khodakhah, 2004). The inhibition of P/Q-type calcium channels abolishes dendritic calcium spikes and induces a switch from regular bursting to tonic firing or irregular bursting of SS spike firing (Womack and Khodakhah, 2004). In the present study, we applied dequalinium, a SK channel blocker, which induced a significant increase in the spontaneous firing rate of SS that was accompanied by a decreased pause of SS and AHP amplitude. Consistent with previous studies (Dunn, 1994; Stocker et al., 1999; Pedarzani et al., 2001; Edgerton and Reinhart, 2003; Womack and Khodakhah, 2004), our results suggested that activation of SK channels was involved in initiation of SS firing discharge in cerebellar PCs *in vivo*.

### CF Discharge Contributes to Spontaneous SS Firing Activity Under *In Vivo* Conditions

In the cerebellar cortex, adult PCs receive excitatory input from a single CF, which is derived from inferior olive cells (Eccles et al., 1966). CF discharge expresses high-frequency bursts, typically consisting of several spikelets (Armstrong and Rawson, 1979; Maruta et al., 2007; Mathy et al., 2009). CF activation evokes CS activity, which plays a critical role in cerebellar cortex behaviors by generating powerful EPSPs onto PCs (Simpson et al., 1996). The CS activity is presumed to represent an important signal for cerebellar cortex functions, conveying timing information from the outside to the cerebellar cortex (Welsh and Llinás, 1997) and triggering parallel fiber-PC synaptic plasticity (Hansel et al., 2001; Ito, 2001). Previous studies demonstrated that the inactivation or removal of the inferior olive induces an increase in spontaneous SS frequency of PCs, whereas reinstating CF input restores tonic levels of SS activity in living animals (Colin et al., 1980; Montarolo et al., 1982; Savio and Tempia, 1985; Cerminara and Rawson, 2004). Consistent with these (Savio and Tempia, 1985; Cerminara and Rawson, 2004), our present results showed that pharmacological blockade of CS activity with an AMPA receptor antagonist significantly increased the frequency of SS firing. Our results indicated that CS activity contributed to inhibiting the spontaneous SS firing rate, suggesting that the information conveyed by CF involved in the modulation of PC output *in vivo* (Cerminara and Rawson, 2004).

In cerebellar slices, the ionic mechanisms of CF activity reduce the frequency of SS discharge (Hounsgaard and Midtgaard, 1989; Miyakawa et al., 1992; Maeda et al., 1999; McKay et al., 2007). CS activity inhibits the SS firing rate, which has been observed in the absence of synaptic inputs, indicating that CF-induced changes in PC output can occur independent of network activation, but dependent on intracellular Ca^2+^ concentrations (McKay et al., 2007). Indeed, the present results showed that either removal of extracellular Ca^2+^ or the chelation of intracellular Ca^2+^ increased the SS firing rate, suggesting that a decreased SS firing rate was dependent on extracellular and intracellular calcium levels of cerebellar PCs *in vivo*. Under *in vitro* conditions, repetitive stimulation of CF has been shown to increase intracellular Ca^2+^ levels and enhance activation of SK channels, resulting in changes in SS firing properties (Hounsgaard and Midtgaard, 1989; Miyakawa et al., 1992; Maeda et al., 1999; McKay et al., 2007). In this study, the PCs were somatic clamping at −70 mV, however, the membrane potential of the dendrites are poorly controlled under *in vivo* conditions (Chu et al., 2011). Thus, CF activation induces membrane depolarization, opening of VDCCs and the subsequent elevation in intracellular calcium triggers SK channel activation. Moreover, the CF-induced strong depolarization could evoke Ca^2+^ influx from CF-PC synaptic NMDARs, which contributes to elevation in intracellular calcium and activating SK channel (Miyakawa et al., 1992; Maeda et al., 1999; Liu et al., 2016). Therefore, the CS activity-induced elevations in intracellular Ca^2+^ are thought to enhance activation of SK channels, thereby increasing AHP generation and setting a lower firing rate of SS (Hounsgaard and Midtgaard, 1989). Results from the present study showed that inhibition of SK channels induced a significant increase in spontaneous SS frequency accompanied by a decreased CS-evoked pause and AHP amplitude. In addition, our results showed that repeated CF stimulation at 1 Hz induced a significant decrease in the spontaneous firing rate of SS, and accompanied with an increase in CV of SS in cerebellar slices, which was also abolished by dequalinium. These results were consistent with previous studies (Hounsgaard and Midtgaard, 1989; McKay et al., 2007), suggesting that activation of SK channels was involved in the CS activity-induced decrease in SS firing rate in cerebellar PCs *in vivo*.

Our results also showed that blockade of GABAergic inhibitory inputs did not significantly affect PC frequency, although blockade of excitatory inputs induced an increase in SS firing rate. This suggested that inhibition of CF inputs contributes to an increased SS firing rate. GCs are relay cells with very low spontaneous activity in the absence of sensory inputs in living animals (Bower and Woolston, 1983; Chadderton et al., 2004; van Beugen et al., 2013). Therefore, mossy fiber-GC-PF excitatory inputs contribute less to SS firing in PCs under anesthetized conditions. Importantly, mossy fiber-GC-PF excitatory inputs are thought to increase the SS firing rate in PCs, and inhibition of these inputs should decrease the SS rate in PCs. Thus, the increased SS firing rate following NBQX treatment was not due to the inhibition of PF excitatory inputs.

### Physiological Significance of CF Discharge Modulates SS Firing Activity

CF activity is assumed to carry information that induces PCs to generate optimal PF input patterns (Ito, 1984). The present results showed that CF input to PCs regulates the SS firing behavior, suggesting that a loss of CF input could result in a fundamental change in spontaneous SS output, as well as deficits in motor coordination (McKay et al., 2007). Indeed, human cerebellar impairments and ataxias in some types of motor learning have been associated with marked atrophy of the inferior olive (Llinás et al., 1975; Martin et al., 1996; Manto, 2005). CF activity also provides widespread Ca^2+^ transients, which are required for PF-LTD induction, and this is assumed to mediate forms of cerebellar motor learning (Ito et al., 1982; Sakurai, 1990; Konnerth et al., 1992; Miyakawa et al., 1992; Ito, 2002). Collectively, spontaneous CS discharge was shown to modulate SS firing activity via activation of SK channels in cerebellar PCs, which further influences cerebellar functions and motor learning in living animals.

## Author Contributions

S-BC and D-LQ: conceived and designed the experiments. X-HJ, H-WW and X-YZ: performed the experiments. C-PC and D-LQ: analyzed the data. Y-ZJ: contributed reagents/materials/analysis tools. X-HJ, H-WW and D-LQ: wrote the manuscript.

## Conflict of Interest Statement

The authors declare that the research was conducted in the absence of any commercial or financial relationships that could be construed as a potential conflict of interest. The reviewer LB and handling Editor declared their shared affiliation, and the handling Editor states that the process nevertheless met the standards of a fair and objective review.

## References

[B1] Armstrong D. M. Rawson J. A. (1979). Activity patterns of cerebellar cortical neurones and climbing fibre afferents in the awake cat. J. Physiol. 289, 425–448. 10.1113/jphysiol.1979.sp012745 458677 PMC1281378

[B2] Barmack N. H. Yakhnitsa V. (2003). Cerebellar climbing fibers modulate simple spikes in Purkinje cells. J. Neurosci. 23, 7904–7916. 12944521 10.1523/JNEUROSCI.23-21-07904.2003 PMC6740591

[B3] Belmeguenai A. Hosy E. Bengtsson F. Pedroarena C. M. Piochon C. Teuling E.. (2010). Intrinsic plasticity complements long-term potentiation in parallel fiber input gain control in cerebellar Purkinje cells. J. Neurosci. 30, 13630–13643. 10.1523/jneurosci.3226-10.2010 20943904 PMC2968711

[B4] Bower J. M. Woolston D. C. (1983). Congruence of spatial organization of tactile projections to granule cell and Purkinje cell layers of cerebellar hemispheres of the albino rat: vertical organization of cerebellar cortex. J. Neurophysiol. 49, 745–766. 6300353 10.1152/jn.1983.49.3.745

[B5] Cerminara N. L. Rawson J. A. (2004). Evidence that climbing fibers control an intrinsic spike generator in cerebellar Purkinje cells. J. Neurosci. 24, 4510–4517. 10.1523/jneurosci.4530-03.2004 15140921 PMC6729399

[B6] Chadderton P. Margrie T. W. Häusser M. (2004). Integration of quanta in cerebellar granule cells during sensory processing. Nature 428, 856–860. 10.1038/nature02442 15103377

[B7] Chen S. Augustine G. J. Chadderton P. (2016). The cerebellum linearly encodes whisker position during voluntary movement. Elife 5:e10509. 10.7554/elife.10509 26780828 PMC4737656

[B8] Chu C.-P. Bing Y.-H. Liu Q.-R. Qiu D.-L. (2011). Synaptic responses evoked by tactile stimuli in Purkinje cells in mouse cerebellar cortex Crus II *in vivo*. PLoS One 6:e22752. 10.1371/journal.pone.0022752 21818384 PMC3144243

[B9] Chu C.-P. Bing Y.-H. Liu H. Qiu D.-L. (2012). Roles of molecular layer interneurons in sensory information processing in mouse cerebellar cortex Crus II *in vivo*. PLoS One 5:e37031. 10.1371/journal.pone.0037031 22623975 PMC3356402

[B10] Colin F. Manil J. Desclin J. C. (1980). The olivocerebellar system. I. Delayed and slow inhibitory effects: an overlooked salient feature of cerebellar climbing fibers. Brain Res. 187, 3–27. 10.1016/0006-8993(80)90491-6 7357475

[B11] Crepel F. Penit-Soria J. (1986). Inward rectification and low threshold calcium conductance in rat cerebellar Purkinje cells. An *in vitro* study. J. Physiol. 372, 1–23. 10.1113/jphysiol.1986.sp015993 2425081 PMC1192747

[B12] Demer J. L. Echelman D. A. Robinson D. A. (1985). Effects of electrical stimulation and reversible lesions of the olivocerebellar pathway on Purkinje cell activity in the flocculus of the cat. Brain Res. 346, 22–31. 10.1016/0006-8993(85)91090-x 3876867

[B13] Dunn P. M. (1994). Dequalinium, a selective blocker of the slow afterhyperpolarization in rat sympathetic neurones in culture. Eur. J. Pharmacol. 252, 189–194. 10.1016/0014-2999(94)90596-7 8157060

[B14] Ebner T. J. (1998). A role for the cerebellum in the control of limb movement velocity. Curr. Opin. Neurobiol. 8, 762–769. 10.1016/s0959-4388(98)80119-0 9914240

[B15] Eccles J. C. Llinás R. Sasaki K. (1966). The excitatory synaptic action of climbing fibres on the Purkinje cells of the cerebellum. J. Physiol. 182, 268–296. 10.1113/jphysiol.1966.sp007824 5944665 PMC1357472

[B16] Edgerton J. R. Reinhart P. H. (2003). Distinct contributions of small and large conductance Ca^2+^-activated K^+^ channels to rat Purkinje neuron function. J. Physiol. 548, 53–69. 10.1113/jphysiol.2002.027854 12576503 PMC2342800

[B17] Grasselli G. He Q. Wan V. Adelman J. P. Ohtsuki G. Hansel C. (2016). Activity-dependent plasticity of spike pauses in cerebellar purkinje cells. Cell Rep. 14, 2546–2553. 10.1016/j.celrep.2016.02.054 26972012 PMC4805497

[B18] Hansel C. Linden D. J. D’Angelo E. (2001). Beyond parallel fiber LTD: the diversity of synaptic and non-synaptic plasticity in the cerebellum. Nat. Neurosci. 4, 467–475. 10.1038/87419 11319554

[B19] Häusser M. Clark B. A. (1997). Tonic synaptic inhibition modulates neuronal output pattern and spatiotemporal synaptic integration. Neuron 19, 665–678. 10.1016/s0896-6273(00)80379-7 9331356

[B21] Hounsgaard J. Midtgaard J. (1989). Synaptic control of excitability in turtle cerebellar Purkinje cells. J. Physiol. 409, 157–170. 10.1113/jphysiol.1989.sp017490 2585289 PMC1190437

[B25] Ito M. (1984). The Cerebellum and Neural Control. New York, NY: Raven Press.

[B22] Ito M. (2001). Cerebellar long-term depression: characterization, signal transduction, and functional roles. Physiol. Rev. 81, 1143–1195. 11427694 10.1152/physrev.2001.81.3.1143

[B23] Ito M. (2002). The molecular organization of cerebellar long-term depression. Nat. Rev. Neurosci. 3, 896–902. 10.1038/nrn962 12415297

[B24] Ito M. Sakurai M. Tongroach P. (1982). Climbing fibre induced depression of both mossy fibre responsiveness and glutamate sensitivity of cerebellar Purkinje cells. J. Physiol. 324, 113–134. 10.1113/jphysiol.1982.sp014103 7097592 PMC1250696

[B26] Jelitai M. Puggioni P. Ishikawa T. Rinaldi A. Duguid I. (2016). Dendritic excitation-inhibition balance shapes cerebellar output during motor behaviour. Nat. Commun. 7:13722. 10.1038/ncomms13722 27976716 PMC5172235

[B27] Kakizawa S. Kishimoto Y. Hashimoto K. Miyazaki T. Furutani K. Shimizu H.. (2007). Junctophilin-mediated channel crosstalk essential for cerebellar synaptic plasticity. EMBO J. 26, 1924–1933. 10.1038/sj.emboj.7601639 17347645 PMC1847665

[B28] Köhler M. Hirschberg B. Bond C. T. Kinzie J. M. Marrion N. V. Maylie J.. (1996). Small-conductance, calcium-activated potassium channels from mammalian brain. Science 273, 1709–1714. 10.1126/science.273.5282.1709 8781233

[B29] Konnerth A. Dreessen J. Augustine G. J. (1992). Brief dendritic calcium signals initiate long-lasting synaptic depression in cerebellar Purkinje cells. Proc. Natl. Acad. Sci. U S A 89, 7051–7055. 10.1073/pnas.89.15.7051 1323125 PMC49643

[B30] Liu H. Lan Y. Bing Y.-H. Chu C.-P. Qiu D.-L. (2016). *N*-methyl-*D*-Aspartate receptors contribute to complex spike signaling in cerebellar purkinje cells: an *in vivo* study in mice. Front. Cell. Neurosci. 10:172. 10.3389/fncel.2016.00172 27445699 PMC4928496

[B32] Llinás R. Sugimori M. (1980). Electrophysiological properties of *in vitro* Purkinje cell dendrites in mammalian cerebellar slices. J. Physiol. 305, 197–213. 10.1113/jphysiol.1980.sp013358 7441553 PMC1282967

[B31] Llinás R. R. Sugimori M. Cherksey B. (1989). Voltage-dependent calcium conductances in mammalian neurons. The P channel. Ann. N Y Acad. Sci. 560, 103–111. 10.1111/j.1749-6632.1989.tb24084.x 2545128

[B33] Llinás R. Walton K. Hillman D. E. Sotelo C. (1975). Inferior olive: its role in motor learning. Science 190, 1230–1231. 10.1126/science.128123 128123

[B34] Loewenstein Y. Mahon S. Chadderton P. Kitamura K. Sompolinsky H. Yarom Y.. (2005). Bistability of cerebellar Purkinje cells modulated by sensory stimulation. Nat. Neurosci. 8, 202–211. 10.1038/nn1393 15665875

[B35] Maeda H. Ellis-Davies G. C. R. Ito K. Miyashita Y. Kasai H. (1999). Supralinear Ca^2+^ signaling by cooperative and mobile Ca^2+^ buffering in Purkinje neurons. Neuron 24, 989–1002. 10.1016/s0896-6273(00)81045-4 10624961

[B36] Manto M. U. (2005). The wide spectrum of spinocerebellar ataxias (SCAs). Cerebellum 4, 2–6. 10.1080/14734220510007914 15895552

[B37] Martin T. A. Keating J. G. Goodkin H. P. Bastian A. J. Thach W. T. (1996). Throwing while looking through prisms. I. Focal olivocerebellar lesions impair adaptation. Brain 119, 1183–1198. 10.1093/brain/119.4.1183 8813282

[B38] Maruta J. Hensbroek R. A. Simpson J. I. (2007). Intraburst and interburst signaling by climbing fibers. J. Neurosci. 27, 11263–11270. 10.1523/JNEUROSCI.2559-07.2007 17942720 PMC6673016

[B39] Mathews P. J. Lee K. H. Peng Z. Houser C. R. Otis T. S. (2012). Effects of climbing fiber driven inhibition on Purkinje neuron spiking. J. Neurosci. 32, 17988–17997. 10.1523/jneurosci.3916-12.2012 23238715 PMC3532857

[B40] Mathy A. Ho S. S. Davie J. T. Duguid I. C. Clark B. A. Häusser M. (2009). Encoding of oscillations by axonal bursts in inferior olive neurons. Neuron 62, 388–399. 10.1016/j.neuron.2009.03.023 19447094 PMC2777250

[B41] McKay B. E. Engbers J. D. T. Mehaffey W. H. Gordon G. R. J. Molineux M. L. Bains J. S.. (2007). Climbing fiber discharge regulates cerebellar functions by controlling the intrinsic characteristics of Purkinje cell output. J. Neurophysiol. 97, 2590–2604. 10.1152/jn.00627.2006 17267759

[B42] Mittmann W. Koch U. Häusser M. (2005). Feed-forward inhibition shapes the spike output of cerebellar Purkinje cells. J. Physiol. 563, 369–378. 10.1113/jphysiol.2004.075028 15613376 PMC1665592

[B43] Miyakawa H. Lev-Ram V. Lasser-Ross N. Ross W. N. (1992). Calcium transients evoked by climbing fiber and parallel fiber synaptic inputs in guinea pig cerebellar Purkinje neurons. J. Neurophysiol. 68, 1178–1189. 1359027 10.1152/jn.1992.68.4.1178

[B44] Montarolo P. G. Palestini M. Strata P. (1982). The inhibitory effect of the olivocerebellar input on the cerebellar Purkinje cells in the rat. J. Physiol. 332, 187–202. 10.1113/jphysiol.1982.sp014409 7153927 PMC1197394

[B45] Naraghi M. Neher E. (1997). Linearized buffered Ca^2+^ diffusion in microdomains and its implications for calculation of [Ca^2+^] at the mouth of a calcium channel. J. Neurosci. 17, 6961–6973. 9278532 10.1523/JNEUROSCI.17-18-06961.1997 PMC6573285

[B46] Ojakangas C. L. Ebner T. J. (1992). Purkinje cell complex and simple spike changes during a voluntary arm movement learning task in the monkey. J. Neurophysiol. 68, 2222–2236. 1491268 10.1152/jn.1992.68.6.2222

[B47] Pedarzani P. Mosbacher J. Rivard A. Cingolani L. A. Oliver D. Stocker M.. (2001). Control of electrical activity in central neurons by modulating the gating of small conductance Ca^2+^-activated K^+^ channels. J. Biol. Chem. 276, 9762–9769. 10.1074/jbc.m010001200 11134030

[B300] Qiu D.-L. Knöpfel T. (2007). An NMDA receptor/nitric oxide cascade in presynaptic parallel fiber-Purkinje neuron long-term potentiation. J. Neurosci. 27, 3408–3415. 10.1523/JNEUROSCI.4831-06.2007 17392457 PMC6672131

[B48] Raman I. M. Bean B. P. (1997). Resurgent sodium current and action potential formation in dissociated cerebellar Purkinje neurons. J. Neurosci. 17, 4517–4526. 9169512 10.1523/JNEUROSCI.17-12-04517.1997 PMC6573347

[B49] Raman I. M. Bean B. P. (1999). Ionic currents underlying spontaneous action potentials in isolated cerebellar Purkinje neurons. J. Neurosci. 19, 1663–1674. 10024353 10.1523/JNEUROSCI.19-05-01663.1999 PMC6782167

[B50] Sakurai M. (1990). Calcium is an intracellular mediator of the climbing fiber in induction of cerebellar long-term depression. Proc. Natl. Acad. Sci. U S A 87, 3383–3385. 10.1073/pnas.87.9.3383 2159149 PMC53904

[B301] Santamaria F. Tripp P. G. Bower J. M. (2007). Feedforward inhibition controls the spread of granule cell-induced Purkinje cell activity in the cerebellar cortex. J. Neurophysiol. 97, 248–263. 10.1152/jn.01098.2005 17050824

[B51] Savio T. Tempia F. (1985). On the Purkinje cell activity increase induced by suppression of inferior olive activity. Exp. Brain Res. 57, 456–463. 10.1007/bf00237832 2984036

[B52] Sceniak M. P. Maciver M. B. (2006). Cellular actions of urethane on rat visual cortical neurons *in vitro*. J. Neurophysiol. 95, 3865–3874. 10.1152/jn.01196.2005 16510775

[B53] Schonewille M. Khosrovani S. Winkelman B. H. Hoebeek F. E. De Jeu M. T. Larsen I. M.. (2006). Purkinje cells in awake behaving animals operate at the upstate membrane potential. Nat. Neurosci. 9, 459–461. 10.1038/nn0406-461 16568098

[B54] Simpson J. I. Wylie D. R. De Zeeuw C. I. (1996). On climbing fiber signals and their consequence(s). Behav. Brain Sci. 19, 384–398. 10.1017/s0140525x00081486

[B55] Stocker M. Krause M. Pedarzani P. (1999). An apamin-sensitive Ca^2+^-activated K^+^ current in hippocampal pyramidal neurons. Proc. Natl. Acad. Sci. U S A 96, 4662–4667. 10.1073/pnas.96.8.4662 10200319 PMC16389

[B57] Swensen A. M. Bean B. P. (2003). Ionic mechanisms of burst firing in dissociated Purkinje neurons. J. Neurosci. 23, 9650–9663. 14573545 10.1523/JNEUROSCI.23-29-09650.2003 PMC6740460

[B58] Usowicz M. M. Sugimori M. Cherksey B. Llinás R. (1992). P-type calcium channels in the somata and dendrites of adult cerebellar Purkinje cells. Neuron 9, 1185–1199. 10.1016/0896-6273(92)90076-p 1281419

[B59] van Beugen B. J. Gao Z. Boele H. J. Hoebeek F. De Zeeuw C. I. (2013). High frequency burst firing of granule cells ensures transmission at the parallel fiber to purkinje cell synapse at the cost of temporal coding. Front. Neural Circuits 7:95. 10.3389/fncir.2013.00095 23734102 PMC3659283

[B60] Vergara C. Latorre R. Marrion N. V. Adelman J. P. (1998). Calcium-activated potassium channels. Curr. Opin. Neurobiol. 8, 321–329. 10.1016/S0959-4388(98)80056-1 9687354

[B61] Welsh J. P. Llinás R. (1997). Some organizing principles for the control of movement based on olivocerebellar physiology. Prog. Brain Res. 114, 449–461. 10.1016/s0079-6123(08)63380-4 9193160

[B302] Witter L. De Zeeuw C. I. (2015). *In vivo* differences in inputs and spiking between neurons in lobules VI/VII of neocerebellum and lobule X of archaeocerebellum. Cerebellum 14, 506–515. 10.1007/s12311-015-0654-z 25735968 PMC4612334

[B63] Womack M. Khodakhah K. (2002). Active contribution of dendrites to the tonic and trimodal patterns of activity in cerebellar Purkinje neurons. J. Neurosci. 22, 10603–10612. 12486152 10.1523/JNEUROSCI.22-24-10603.2002 PMC6758439

[B62] Womack M. D. Khodakhah K. (2004). Dendritic control of spontaneous bursting in cerebellar Purkinje cells. J. Neurosci. 24, 3511–3521. 10.1523/jneurosci.0290-04.2004 15071098 PMC6729759

[B64] Wulff P. Schonewille M. Renzi M. Viltono L. Sassoè-Pognetto M. Badura A.. (2009). Synaptic inhibition of Purkinje cells mediates consolidation of vestibulo-cerebellar motor learning. Nat. Neurosci. 12, 1042–1049. 10.1038/nn.2348 19578381 PMC2718327

